# Efficient simulation strategy to design a safer motorcycle

**DOI:** 10.1007/s11044-023-09879-8

**Published:** 2023-02-07

**Authors:** Steffen Maier, Jörg Fehr

**Affiliations:** Institute of Engineering and Computational Mechanics, University of Stuttgart, Pfaffenwaldring 9, 70569 Stuttgart, Germany

**Keywords:** Multibody system, Nonlinear finite elements, Crashworthiness, Passive safety, Impact biomechanics

## Abstract

This work presents models and simulations of a numerical strategy for a time and cost-efficient virtual product development of a novel passive safety restraint concept for motorcycles. It combines multiple individual development tasks in an aggregated procedure. The strategy consists of three successive virtual development stages with a continuously increasing level of detail and expected fidelity in multibody and finite element simulation environments. The results show what is possible with an entirely virtual concept study—based on the clever combination of multibody dynamics and nonlinear finite elements—that investigates the structural behavior and impact dynamics of the powered two-wheeler with the safety systems and the rider’s response. The simulations show a guided and controlled trajectory and deceleration of the motorcycle rider, resulting in fewer critical biomechanical loads on the rider compared to an impact with a conventional motorcycle. The numerical research strategy outlines a novel procedure in virtual motorcycle accident research with different levels of computational effort and model complexity aimed at a step-by-step validation of individual components in the future.

## Introduction

**Fig. 1:**
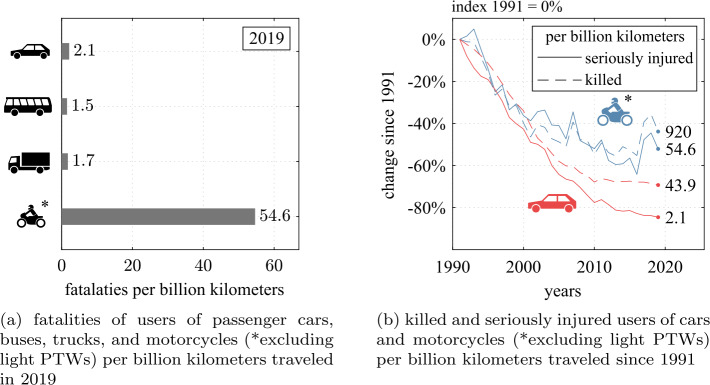
Road-traffic fatalities per kilometer traveled in Germany [9, 10] (Note: Figures for 2020 are also available, but according to [9], the mileage for 2020 is subject to great uncertainty due to the coronavirus pandemic)

A motorcycle accident is a very complex event where the rider and the motorcycle interact with many environmental factors. Due to the exposed position of the riders, the vehicle itself does not provide protection to the rider in the event of a collision with an accident opponent or a roadside structure. Instead, violent ejection of the rider from the motorcycle is a likely accident pathway when the vehicle suddenly comes to a standstill [1–3]. When striking objects in their path and the ground, the consequences are often severe or fatal for the involved riders. This results in an excessive risk that motorcycles are 25 times more deadly per kilometer traveled than passenger cars, see Fig. 1a. Accident analyses of severe or fatal injuries [4] show that the personal protective equipment worn is insufficient to mitigate the consequences of serious accidents, which motivates the first research question *RQ1: Would it be safer to be restrained to a motorcycle?* This will be investigated with a motorcycle with a novel safety concept where the rider is restrained by thigh belts, multiple airbags, and leg impact protection. The concept’s goal is to guide the rider’s impact trajectory for controlled deceleration and to prevent direct impacts on opposing vehicle structures.

A safety system that effectively protects the rider must function robustly in the many possible accident scenarios. The possible scenarios for accidents involving motorcycles are very diverse. Because of their complex nature, it is very difficult to find passive safety solutions that are effective in as many accident configurations as possible and that at least have neutral or nondetrimental effects on the accident consequences in off-design accident scenarios. This results in many design iterations and cannot be achieved with costly experimental methods alone. Therefore, the merits of virtual methods, like multiquery and multiparameter design studies, are needed. It includes procedures that allow for a systematic approach to the design of the proposed passive safety systems and the investigation of their protective performance in representative accident scenarios. To test many different solutions efficiently, models with varying levels of complexity and computing effort that capture different aspects of the accident behavior are necessary, which leads to the main subject of this work, the second research question *RQ2: What virtual models and methods meet the above requirements to investigate many possible design iterations and accident configurations effectively and efficiently?* For this, this work presents a virtual simulation-based product design procedure consisting of three successive development stages with a continuously increasing level of detail and expected fidelity.

The novelty of this work is the modeling and simulation strategy for crashworthiness investigations of a motorcycle with a systematic model generation approach with different levels of computational effort and model complexity. It outlines a novel procedure in virtual motorcycle accident research and passive safety equipment development that is very similar to common strategies in the development process of passenger cars for occupant protection.

First, this work describes the aims and concept of the novel safety concept. It briefly introduces the essential computational models in current crashworthiness investigation, identifying and substantiating the choice for the modeling and simulation strategy. An emphasis of this work is on describing the modeling of the vehicle, passive safety systems, and rider surrogates. The results focus on a meaningful description of the operating principles and their influence on accident behavior based on the virtual models. The advantages of the individual modeling stages are discussed.

### Novel safety concept for motorcycles

Powered two-wheelers (PTWs), or more commonly called *motorcycles*, are a popular means of transportation. They are attractive as a relatively cheap, agile, and space-saving mobility solution. Annual new registrations of motorcycles and mopeds, for example, in Europe had a general positive trend from 2013 to 2019 [5, 6] and many European countries exceeded pre-pandemic levels in 2021 [7]. However, because of their low level of passive protection, they carry a disproportionate risk of severe or fatal injury for their riders in accidents compared to cars. The World Health Organization estimates that every year about 375,000 users of two and three-wheelers die in road traffic accidents [8]. This is 28% of all road traffic deaths. Only fatalities of riders and passengers of four-wheeled vehicles with a share of 29% were marginally more frequent, although these are used far more. A more detailed analysis of reported accidents in Germany illustrates the immense risk. As shown in Fig. 1a by accounting for the mileage for motorcycles (excluding light PTWs, such as mofas and mopeds), the risk of a fatal accident is more than 25 times higher than for cars, buses, or trucks, individually—the trend since 1991 in Fig. 1b illustrates that the risk of a serious injury with 920 vs. 43.9 serious injuries per billion kilometers is likewise much higher and the safety of motorcycles has not improved nearly as much as that of passenger cars in the past decades.

**Fig. 2:**
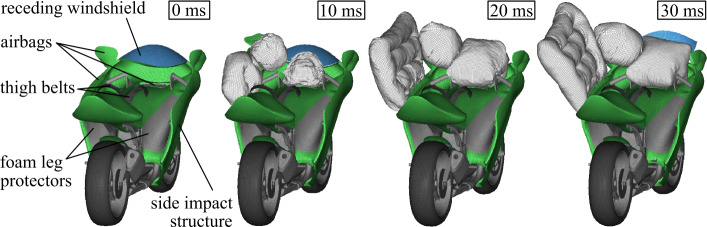
Safety systems of the novel safety concept for motorcycles (Note: The right airbags are not displayed). A video of the safety concept is shown in [11]

The proposed novel safety concept shown in Fig. 2 aims to improve rider passive safety in accidents significantly. It consists of a newly designed motorcycle body, seat belts, multiple surrounding airbags, foam leg impact protectors, and side impact structure. The concept envisages that in the event of an accident, the rider is restrained to the motorcycle by two thigh belts. This causes the rider’s upper body to rotate around the belt restraint. The surrounding airbags then decelerate the upper body rotation in a controlled manner and protect the rider from hard contact with an accident opponent, the road, or road-side structures. The foam leg impact protectors absorb the impact of the legs on the motorcycle cockpit, and the side impact structure protects the lower extremities laterally. The lateral fairing is shaped so that comfortable mounting, riding, and balancing while stationary should still be possible—which must be proved via an ergonomic analysis—while retaining the advantages of a motorcycle as a compact means of transportation.

### Computational models for crashworthiness

**Fig. 3:**
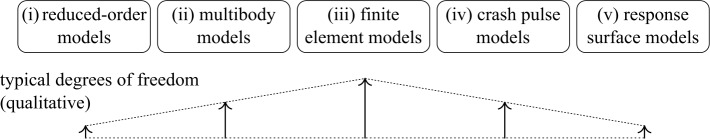
Categories of mathematical models for investigating crashworthiness

A recent review on mathematical models to assess vehicle impact crashworthiness [12] classifies existing models into the five model categories shown in Fig. 3. (i) Reduced-order models include lumped parameter models and commonly replicate vehicle impacts with lumped masses in interaction through simple mechanical elements such as springs and dampers. They aim to capture the fundamental impact kinematics, often for unidirectional response with very few degrees of freedom. (ii) Multibody (MB) models in crashworthiness are usually more complex systems of rigid and flexible bodies, constrained by idealized joints and loaded by internal and external force elements. In generalized coordinates, the equation of motion for a holonomic MB system is

1 $$ \boldsymbol{M}(\boldsymbol{y},t) \cdot \boldsymbol{\ddot{y}} = \boldsymbol{k}_{\mathrm{c}}(\boldsymbol{y},\boldsymbol{\dot{y}},t) + \boldsymbol{k}_{\mathrm{e}}(\boldsymbol{y},\boldsymbol{\dot{y}},t), $$

where $\boldsymbol{M} \in \mathbb{R}^{f \times f}$ is the global mass matrix, $\boldsymbol{\ddot{y}} \in \mathbb{R}^{f \times 1}$ is the acceleration vector, $\boldsymbol{k}_{\mathrm{c}} \in \mathbb{R}^{f \times 1}$ is the vector of the generalized Coriolis and centrifugal forces, and $\boldsymbol{k}_{\mathrm{e}} \in \mathbb{R}^{f \times 1}$ is the vector of the generalized applied forces. In addition, contact interactions of the bodies are often implemented via nonlinear contact characteristics also representing the deformation of the rigid bodies. A three-dimensional system of $p$ rigid bodies with $r$ holonomic, rheonomic constraints has a number of degrees of freedom of $f=6p-r$ [13]. MB models can be used for kinematic and kinetic analysis, including the structural response of vehicles [14] and the prediction of kinematic response [15] and biomechanical loading [16] of humans. (iii) Finite element (FE) methods in crashworthiness deal with geometry, material, constraint, and contact nonlinearities, making solving such models more difficult and computationally costly. The discretized equations of motion of an FE model with a Lagrangian mesh can be written as

2 $$ \boldsymbol{M} \cdot \boldsymbol{\ddot{q}} = \boldsymbol{f}^{ \mathrm{int}} - \boldsymbol{f}^{\mathrm{ext}}, $$

where $\boldsymbol{M} \in \mathbb{R}^{n \times n}$ is the mass matrix and $\boldsymbol{\ddot{q}} \in \mathbb{R}^{n \times 1}$ are the nodal accelerations that result from the internal forces $\boldsymbol{f}^{\mathrm{int}} \in \mathbb{R}^{n \times 1}$, representing element stresses, and external forces $\boldsymbol{f}^{\mathrm{ext}} \in \mathbb{R}^{n \times 1}$ from boundary conditions, such as acceleration fields, constraints, or contacts [17]. Depending on the element formulation, each node has different degrees of freedom, commonly three or six. The models accurately represent complex geometries and incorporate diverse material models in the detailed representation of vehicles and human surrogates. They are particularly applied to capture vehicle structural behavior and localized effects such as detailed injury mechanisms in human body models. (iv) Crash pulses models are substructure approaches [18] where the vehicle impact response is substituted, e.g., from experiments or full vehicle simulations [19]. It reduces the degrees of freedom of high fidelity models by strategically replacing constituents with lower fidelity physical surrogate models. (v) As its mathematical equivalent, response surface models are mathematical surrogate models with usually even fewer degrees of freedom. They describe relationships between very few input and output variables obtained by various mathematical and statistical methods. These models aim to predict and improve the behavior of complex designs with minimized computational effort.

Although not remotely comparable to crash safety of passenger car occupants, there have been efforts to model motorcycle crashworthiness. A rare reduced order model of motorcycles is in [20]. There are several MB models of motorized two-wheelers to model car impacts [21, 22], road-side barrier impacts [23–25], and solo accidents [26]. There are also published works on detailed FE models of various motorcycles types, such as sports and sport touring motorcycles [27, 28], a large touring motorcycle [29], or a tilting three-wheeled scooter [30]. However, there is a lack of reduced models to efficiently investigate isolated aspects of motorcycle or motorcyclist crash behavior in submodel approaches. Also, there is a lack in combinations of two or more models, e.g., of different detailing and complexity, into a connected workflow. This has long been state-of-the-art in accident simulation of cars.

## Modeling

### Modeling and simulation strategy

Common methods and strategies used in the vehicle development process of passenger cars for occupant protection break down the accident event into several individual problems, see, e.g., [31]. Thus, in experiments, the performance of the components, the interaction of the vehicle structure with the accident opponent, and the occupants’ behavior within the vehicle interior are often considered separately. Individual components or subassemblies are subjected to separate component tests to assess the functionality of the individual systems and the structural capacity of a component, substructure, and vehicle body. The interaction of the restraint systems with mechanical human surrogates, anthropometric test devices (ATDs), as vehicle occupants is optimized in sled tests. In these experiments, reinforced partial vehicle bodies represent the relevant vehicle’s interior to investigate the effect of the passive safety systems without destroying an entire vehicle. Full-vehicle laboratory crash tests of new products are rarely carried out during the design process but rather at the very end of the development to ultimately prove the occupant protection for vehicle approval or to evaluate occupant safety for consumer ratings. Similarly, in the virtual vehicle development process, there are multiple mathematical model representations used for individual design aspects, see Sect. 1.1. Corresponding to the experimental procedure mentioned above, the depth of reproduction in occupant safety studies is often reduced not to simulate the entire system. Thus, only the vehicle structures are considered for the reconstruction of the vehicle interaction and dimensioning of the deformation and contact structures. The occupant restraint interaction and biomechanical loading is simulated using crash pulses and vehicle interior models.

**Fig. 4:**
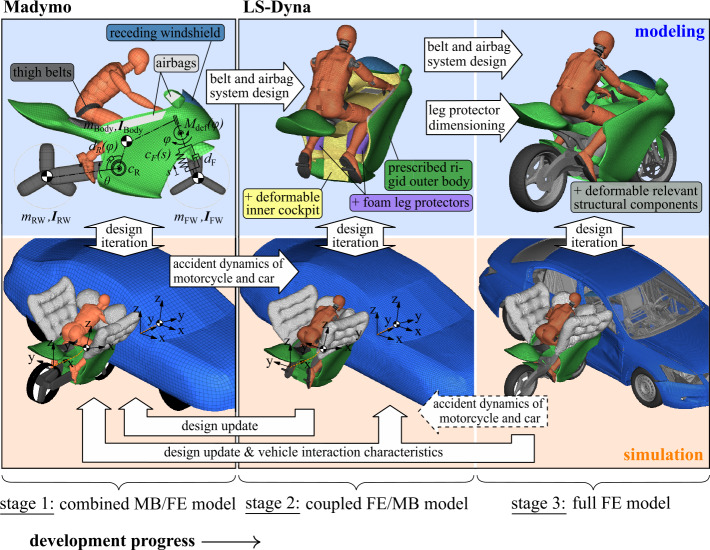
Modeling and simulation strategy

Such a breakdown, both for experiments and simulations, is usually not possible when investigating accidents involving current conventional motorcycles, e.g., in collisions with passenger cars. Here, the rider interacts with the motorcycle, opposing vehicle structures, and road and road-side structures. A safety concept that restrains the motorcyclist to the motorcycle and isolates it from the accident environment not only has the advantage of potentially improving passive safety, which must be demonstrated, but also allows to apply similar modeling and simulation strategies for a more systematic investigation. In Fig. 4 such a design strategy is developed. It consists of three subsequent development stages with a continuously increasing level of detail:

- Stage 1: The motorcycle as well as a rider surrogate are modeled in a combined MB and FE approach in the Madymo software environment (version MADYMO 2020.1), as the authors introduced in [32]. The vehicles are MB systems with joint restraint and contact characteristics based on simulation models of full-scale crash tests of conventional motorcycles and fitted to the experience from the full FE representations (stage 3). The multiple airbags and the thigh belts are modeled with 1D and 2D FE elements using the FE capabilities of the Madymo software environment. This stage features few degrees of freedom and associated low numerical costs while capturing the essential physics of the impact. It is used to initially tune and improve the safety system.
- Stage 2: The rider interaction surfaces of the motorcycle cockpit are modeled further detailed as an FE model in the LS-Dyna software environment (version LS-DYNA R9.3.1 MPP), as the authors developed and showed in [33, 34]. The modeling and parameterization of the airbags’ inflation and thigh belts’ pretensioning and load-limiting is equivalent to the MB simulations. The FE rider interaction model represents deformable cockpit surfaces that include foam impact protectors, which the authors characterized in [35]. To replicate the crash kinematics and vehicle intrusion, the motorcycle and car are (offline-)coupled to a crash pulse. Prescribed motions represent the multiaxial rigid body motions from the MB simulations where the car’s body geometry acts as reaction surfaces for the airbags. This stage allows using state-of-the-art FE HBMs as rider surrogates, e.g., to examine injuries on the tissue level, while reducing numerical costs by using prescribed vehicle motions.
- Stage 3: The motorcycle, the already tuned passive safety systems equivalent to stage 1 and 2, the rider surrogate, and an accident opponent are modeled as full FE representations in Ls-Dyna. The motorcycle’s structurally relevant components that determine the crash behavior are deformable. The front and rear suspension, rotating wheels, and front fork steering are modeled with kinematic joints. This stage allows modeling the vehicle response with a high degree of detail to investigate the deformation characteristics of the motorcycle itself and the structural interaction with opposing vehicles. It is used to predict the performance of the finalized design accurately. The resulting vehicle interactions are used to tune the rigid body interaction of the stage 1 model.

The procedure is divided into *modeling* and *simulation*, in between design iterations and updates from model validation are carried out. As shown in this work, the development progress follows a sequence from left to right, with stage 3 as the final stage. Linking design updates for the motorcycle structure and passive safety systems to the lower stages results in design loops through stage 1 and 2 to update and maintain an identical design with uniform design parameters in all the simulation stages. However, the sequence could also be modified. Thus, the vehicle kinematics from the full FE vehicle interactions could be applied to the stage 2 model, as indicated with the dashed arrow.

### Vehicles

#### Multibody systems

**Fig. 5:**
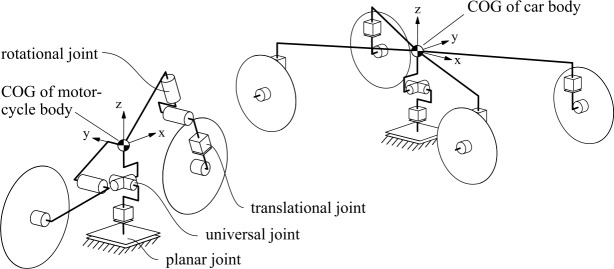
Kinematic structure of the motorcycle and accident opponent

**Fig. 6:**
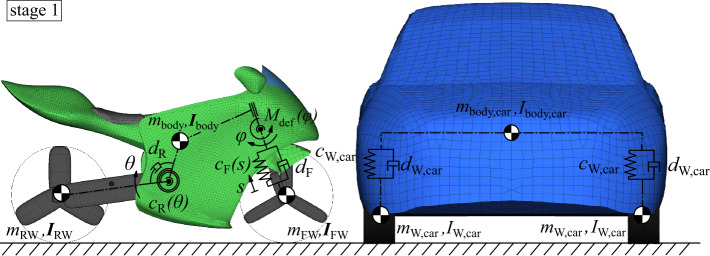
Joint restraint characteristics of the motorcycle and accident opponent

**Fig. 7:**
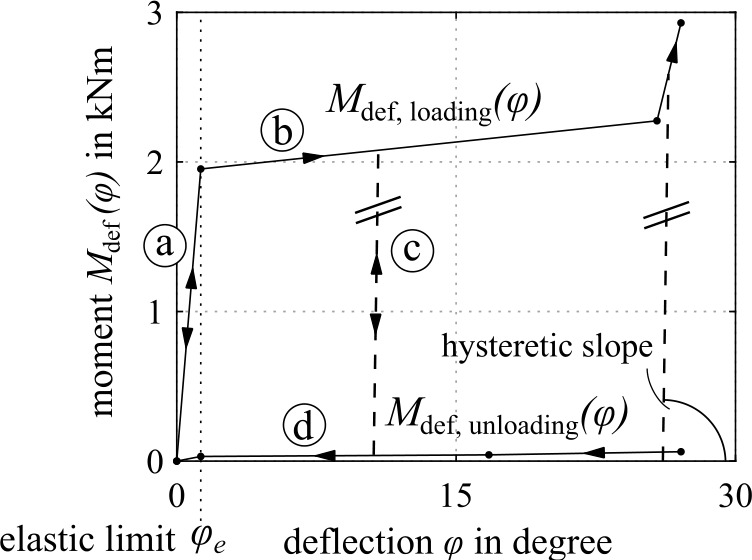
Loading and unloading characteristics $M_{\mathrm{def}}( \varphi )$ of the telescopic front fork impact deformation

To replicate the crash dynamics of the motorcycle and an opposing vehicle, a passenger car, the main parts that determine their significant crash behavior, are modeled as rigid bodies. They are connected with kinematic joints as schematically illustrated in Fig. 5 with restraint characteristics as shown in Fig. 6. The bodies are the wheels and the vehicle’s body each, which are specified by their geometry with ellipsoid and facet surfaces, mass $m$, and inertia $\boldsymbol{I}$. The kinematic structure replicates (i) rotating wheels, (ii) front and rear suspension, (iii) front fork steering, and (iv) front fork impact deformation. The suspension is coupled by nonlinear spring restraints $c_{ \mathrm{FW}}(s)$ and $c_{\mathrm{RW}}(\theta )$ with front suspension deflection $s$ and rear suspension deflection angle $\theta $, and constant dampening restraints $d_{\mathrm{FW}}$ and $d_{\mathrm{RW}}$. A rotational joint couples the front telescopic fork for frontal impact response, representing reward fork deformation. Loading and unloading response via moment $M_{ \mathrm{def}}(\varphi )$ from deflection angle $\varphi $, shown in Fig. 7, allows for energy dissipation through hysteresis. It represents linear response up to the elastic limit $\varphi _{\mathrm{e}}$ () and beyond plastic deformation along $M_{\mathrm{def,loading}}( \varphi )$ (). Unloading is first parallel to the hysteresis slope () and then along $M_{ \mathrm{def,unloading}}(\varphi )$ (). Reloading while unloading follows that path until reaching $M_{ \mathrm{def,loading}}(\varphi )$. The incorporated Madymo contact interactions characteristics are similar hysteretic models with load functions of penetration depths of the contacting ellipsoid and facet surfaces. For a multibody motorcycle model with equivalent kinematic structure, see [21]. Besides, [36] describes a similar model without front fork deformation.

The opposing vehicle is a 2011 Honda Accord, a four-door passenger sedan with a mass of 1,668 kg, an overall height of 141 cm, and vehicle parameters according to [37].

**Fig. 8:**
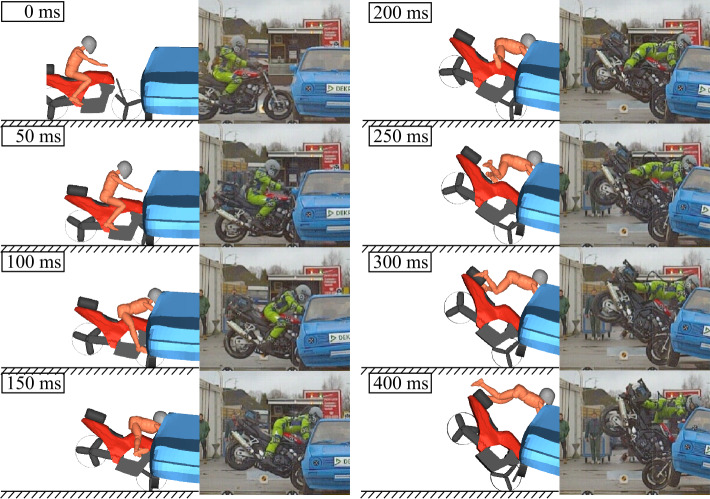
MB simulation of full-scale crash test SH01.01 [38] with a conventional motorcycle Yamaha FZS 600 Fazer and a helmeted Hybrid III 50^th^ ATD against a VW Golf II

**Fig. 9:**
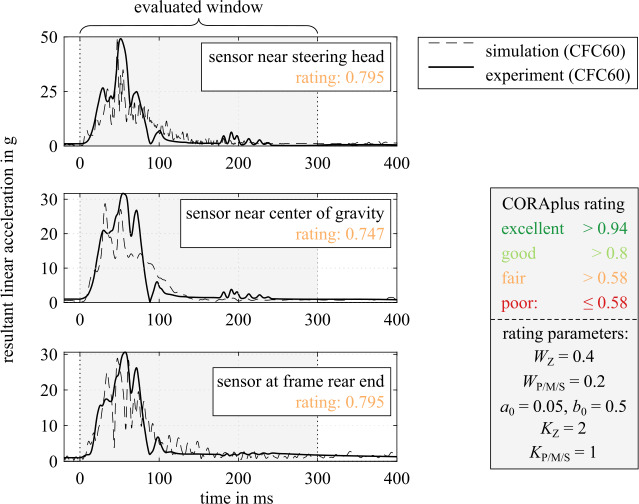
Resultant linear motorcycle accelerations at multiple locations of MB simulation of full-scale crash test SH01.01 compared to experimental sensor data

The given front fork deflection and contact characteristics are based on manually fitted simulations of full-scale crash tests of conventional motorcycles and a Hybrid III 50^th^ ATD against passenger cars from Dekra [38]. For a complete discussion of the simulations of conventional motorcycles with riders, see [32]. A comparison of motion sequence and motorcycle acceleration sensor data for a frontal impact of a Yamaha FZS 600 Fazer impacting at 48.5 km/h at a right angle into the side of the stationary car is given in Fig. 8 and Fig. 9. The accelerations are filtered with a CFC filter (CFC for channel frequency class) with filter class 60, see [39]. The motorcycle and car trajectories and accelerations conform well, but similar helmet impact location at the car could not be achieved with the MB approach. This is because in the MB simulation the initial suspension compression of the car at the impact side (see snapshot at 100 ms) does not correlate. The real vehicle from the crash test has a softer door and stiffer door sill area, which likely causes the vehicle to roll to the impact side initially; see also Fig. 26. An approximation as a homogeneous body in the MB approach is not sufficient here. In a future step, feedback from stage 3 simulations could help to create a more fitting/better multibody model of the opposing vehicle.

To quantitatively compare the motorcycle accelerations, the CORA (CORelation and Analysis) objective rating [40] is used. It offers an objective metric for the correlation of time history signals by combining two independent methods: a corridor rating and a cross-correlation rating. Using the stipulated default parameters for weights $W$, corridor widths $a_{0}$ and $b_{0}$, and rating exponents $K$ of the rating method (version CORAplus Release 4.0.4 [41]), the CORA rating the agreement for the primary impact (0-300 ms) is in the very upper range of *fair*, very near *good*. The used rating parameter values are given in Fig. 9.

#### Coupled finite element and multibody model

**Fig. 10:**
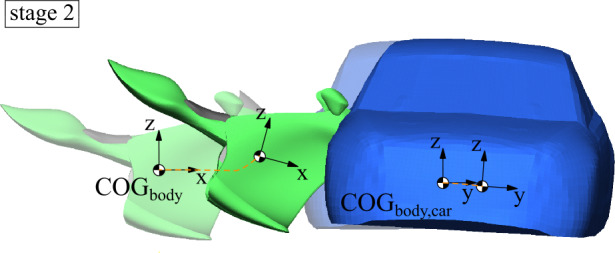
Coupled FE rider interaction model with rigid body trajectories from MB simulation

An FE model represents the rider interaction surfaces and the passive safety equipment for a further detailed step in the overall simulation strategy. 3D multiaxial rigid-body trajectories from the linear and angular motion from the MB simulations replicate the vehicle motion for crash dynamics by prescribing the outer body geometry of the motorcycle, see Fig. 10. For translational motion, the initial linear velocities and the time histories of linear acceleration are used, and for angular motion, the velocities are used. The safety concept envisages that when deployed, the side airbags interact with the opposing vehicle structures or the ground. Therefore, the prescribed outer surface of the accident opponent represents the reaction surface. The overall procedure corresponds to experiments or simulations of vehicle occupants in which impact pulses also reproduce the accident interaction with an accident opponent.

#### Full finite element models

**Fig. 11:**
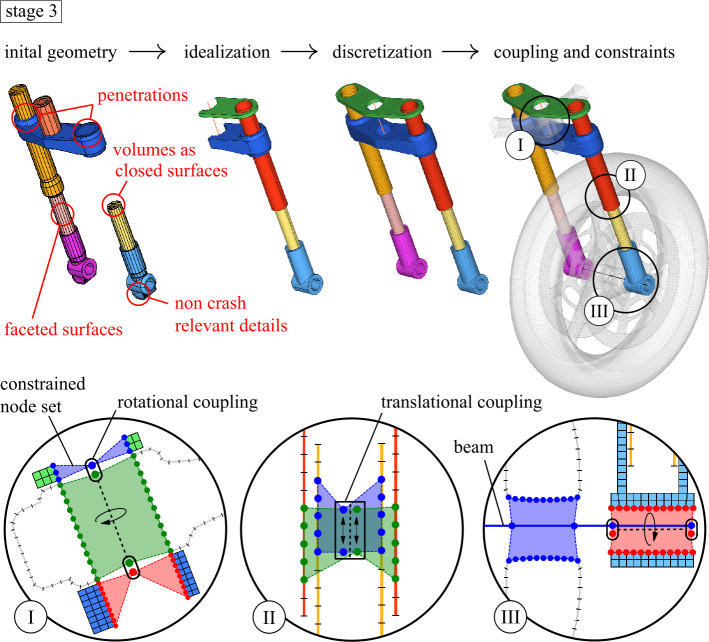
FE modeling pre-processing workflow with joint coupling details

For the highest degree of detail, a full FE representation of the motorcycle is developed from a concept design CAD model that represents the main construction features of the motorcycle. The CAD model comprises standard–“off the shelf”—components for motorcycles from various model libraries and many custom parts. For investigations of crashworthiness, the model aims to represent the interaction with the crash opponent, structural loading and deformation, and energy absorption of the motorcycle structure. As a result, the model focuses on representing the crash-relevant and structural components, i.e., the front wheel, tire, and suspension assembly. Parts that are assumed not to deform because they are very stiff or outside of crash deformation, such as drivetrain components, are modeled rigid, but their dimensions, weight, and position are considered. Figure 11 illustrates the steps performed on the way to the discretized, coupled system using the telescopic front fork assembly as an example. It consists of cleaning up and simplifying the CAD geometry due to the initially relatively low model quality mentioned above and is followed by the discretization and connection of the components, as shown in section cuts of some revolute and translational joint couplings below. A unique feature of the motorcycle structure is a rigid foam crash box in the cockpit nose, which aims for controlled energy transfer to prevent rollover in a frontal impact and elevated side impact structures that protect the lower extremities laterally. The model consists of 148 parts from 377,000 elements with 323,000 nodes. It has seven kinematic joints: front wheel rotation (2), telescopic front fork suspension (2), rear-wheel rotation, front fork steering, and rear swing arm rotation.

The opposing vehicle, an FE model of the 2011 Honda Accord four-door passenger sedan, is chosen from the publicly available vehicle model database of the National Highway Traffic Safety Administration (NHTSA) [37, 42].

### Rider restraint

#### Thigh belt modeling

**Fig. 12:**
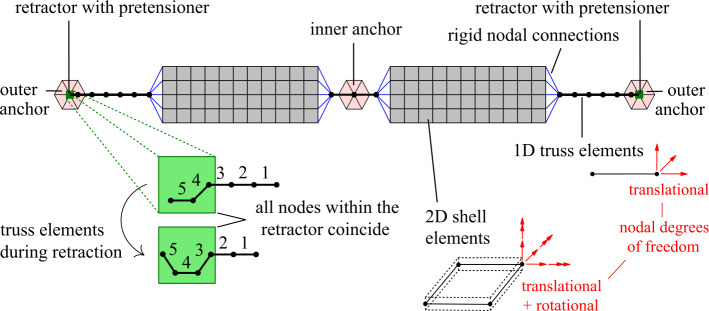
Structure of 1D truss and 2D membrane hybrid mesh belt with combined pretensioning and retracting capabilities

The structure of the belt system is schematically illustrated in Fig. 12. The two belt sections are four-node 2D shell and two-node 1D truss elements. The truss nodes have only translational nodal degrees of freedom, carrying only in-line loads. The shell nodes have translational and rotational degrees of freedom, transmitting forces and moments. The belts are connected at both ends to the attachment points to the motorcycle body (anchors). At their outer ends, nonlinear retractors with pre-tensioning and load-limiting are implemented to control the belt force $f(t)$ by manipulating the belt length $l(t)$ during impact. The retractor removes any pre-tension or initial slack in the belt by supplying or retracting belt length. After the activation or *firing*, a pre-tensioning regime is determined by a function of belt pay-in vs. time

3 $$\begin{aligned} L_{\mathrm{pay-in}}(t)= \textstyle\begin{cases} 0, \quad & \textrm{if} \; t < t_{\mathrm{fire}} \\ -\frac{100}{15} \frac{\textrm{mm}}{\textrm{ms}}(t-t_{\mathrm{fire}}), \; & \textrm{if} \;t_{\mathrm{fire}} \leq t \leq t_{\mathrm{fire}}+15 \; \mathrm{ms} \\ -100 \; \textrm{mm}, \quad & \textrm{if} \;t > t_{\mathrm{fire}}+15 \; \mathrm{ms} \end{cases}\displaystyle . \end{aligned}$$

Simultaneously, the force level at the outer belt ends is limited by the load-limiter to $F_{\mathrm{load-limit}}=$ 2 kN in the connected truss element by giving out belt material. The firing of the systems is set to a fixed time of $t_{\mathrm{fire}}=$12 ms after an initial accident contact. This follows findings of sensor concepts for accident detection for motorcycles in [43], i.e., based on front fork deceleration and specifications of the Honda Gold Wing sensor system in [44], the only motorcycle to date that is equipped with a standard, although only optional, airbag introduced in 2006 [45]. The retractors with belt tensioners are currently modeled as point elements, i.e., the packaging is currently not accurately considered.

**Fig. 13:**
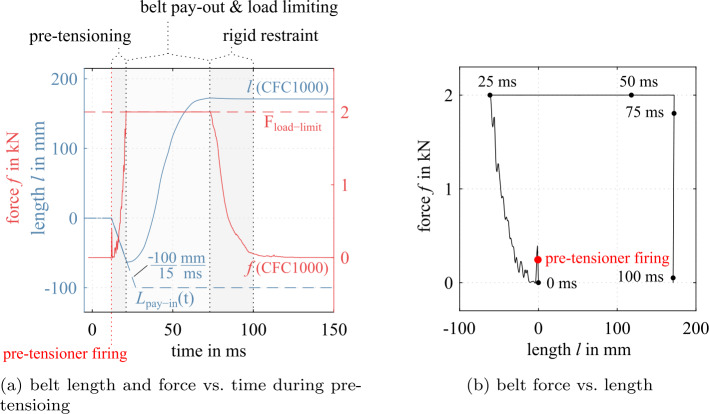
Belt end force and length during pretensioning and load limiting in a frontal impact

As given in Fig. 13a and b for a frontal crash scenario with a motorcycle impact speed of 48.5 km/h against a stationary car (same as in Fig. 8), the force and belt length response can be divided into three consecutive phases: (i) pretensioning, (ii) belt pay-out because of load limiting, and (iii) a rigid restraint without any belt in- or output. Here, pre-tensioning reduces the frontal displacement of the rider in frontal collisions through early intervention of the belt restraint and provides a constant force level, thus reducing peak forces and peak deceleration of the rider. Apart from initial belt routing, the design variables are the belt geometry, such as the width, the positions of the attachment points, the belt pay-in function, and the belt load limit.

#### Airbag modeling

The three types of airbags of the safety concept are single chamber 2D and 3D designs with and without internal structure and with and without exhaust openings. For example, the front airbag is a 3D design, meaning that its geometry in its undistorted reference or design configuration encloses a volume compared to a flat 2D design. The frontal airbag has a wedge shape, no internal structures, and two exhaust openings to regulate the airbag pressure and, consequently, the rider’s deceleration when impacting. The windshield is designed to fail under contact pressure from the expanding front airbag structurally.

**Fig. 14:**
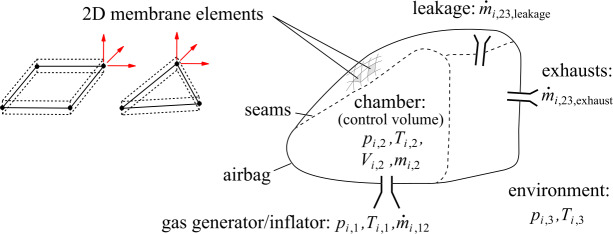
Concept of the uniform pressure method (UPM)

There are multiple methods to model inflation of an airbag, see overviews in [46, 47]. These are uniform pressure concepts [48] and methods that discretize the gas flows using mesh-based [49] or particle-based [50] approaches. The latter have advantages in reproducing the beginning of the airbag expansion. Therefore, these are especially important for more fundamental out-of-position investigations. In this work, the uniform pressure method (UPM) is sufficient and an appropriate choice as an investigation in the context of the early development phase. As shown in Fig. 14, the airbag is discretized with three- and four-node membrane elements, since bending stiffness is often assumed negligible for simulating airbag fabrics. Expansion is determined by entering and exiting gas mass flows $\dot{m}$ through the inflator, airbag exhausts, and airbag seam and fabric leakage. The mass balance complies for each time step $i$ such as

4 $$\begin{aligned} \dot{m}_{i,\mathrm{tot}} & = \dot{m}_{i,\mathrm{in}} + \dot{m}_{i, \mathrm{out}} \\ & = \dot{m}_{i,12} + \dot{m}_{i,23\mathrm{,exhaust}} + \dot{m}_{i,23 \mathrm{,leakage}}. \end{aligned}$$

The internal airbag chamber variables are calculated from scalar thermodynamic equations. At the beginning of each time step, the volume of the expanding airbag chamber $V$ is determined. In the basic principle, assuming uniform pressure $p$ and temperature $T$ of an ideal gas in the adiabatic airbag chamber, the pressure is expressed as

5 $$ p_{i} = (\gamma -1) \; \rho _{i} \; e_{i}, $$

where $\gamma $ is the isentropic coefficient $c_{p}/c_{v}$, $\rho $ is the density, and $e$ is the specific internal energy. The dependency for two neighboring steps is

6 $$ \frac{e_{i}}{e_{i-1}} = \left (\frac{V_{i-1}}{V_{i}}\right )^{\gamma }+ \left (\frac{\rho _{i}}{\rho _{i-1}}\right )^{\gamma}. $$

From volumes $V_{i-1}$, $V_{i}$, and specific internal energy $e_{i-1}$ of the previous and current steps, the current specific internal energy $e_{i}$ is computed. With Eq. (5), this leads to the pressure and ultimately to the normal force acting on the airbag fabric.

**Fig. 15:**
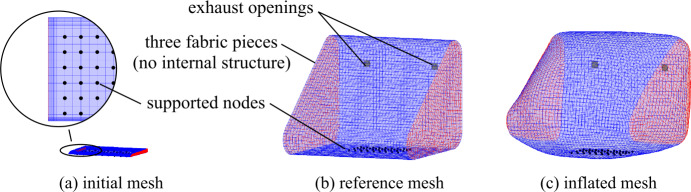
Front airbag meshes for initial metric method (a)–(b) and inflated mesh (c)

To define the initial geometry of an airbag chamber, there are methods of scaling and more complex folding [51, 52]. Here, scaled initial meshes and the so-called initial metric methodology (IMM) [53] are used to model the expanding control volume. This method uses two meshes, an initial mesh and an undeformed reference or design mesh, see Fig. 15. Due to scaling, the initial mesh has highly distorted elements. An algorithm calculates internal forces based on the geometric differences between the initial and the reference mesh and allows the elements to relax to their reference surface area. It circumvents considerably more complex folding procedures, which is usually a laborious and time-consuming task, especially in tight packaging, and detrimental in the initial stages of product development. Using these methods, the design variables for deployment, apart from the fabric’s implemented material model, include the airbag’s external and internal geometry, a mass inflow function with constant inflow temperature, and the exhaust hole area.

**Fig. 16:**
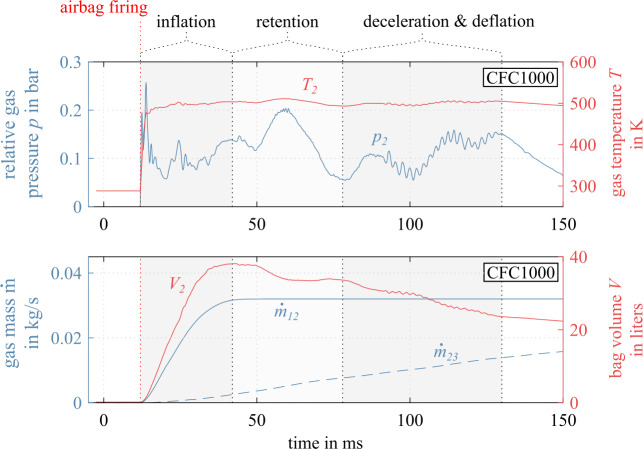
Gas generator and airbag chamber variables of front airbag in a frontal impact

Resulting inflation variables in a frontal impact are given in Fig. 16. Again, the operation can be divided into multiple consecutive phases: after firing (i) inflation because of gas generator input, followed by (ii) retention of airbag volume and pressure until (iii) rider impact and controlled rider deceleration, where the gas deflates from the chamber through the exhausts.

#### Leg impact protection modeling

**Fig. 17:**
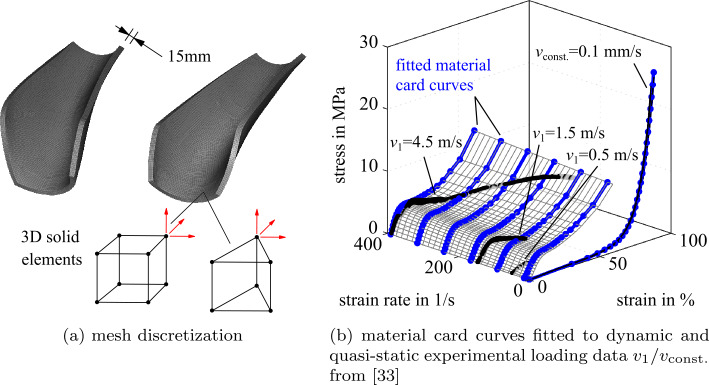
Leg protector modeling

The 15 mm foam impact protection, shown in Fig. 17a, is modeled using eight-node (hexahedron) and six-node (prism) 3D solid elements (ELFORM = −2; fully integrated elective reduced solids). It provides a soft surface while achieving good impact energy absorption. The designated material, an energy absorbing high-density foam (425 g/l), has an impact-rate-dependent material behavior: on fast impacts, it responds very firm, while under slower loading, it remains soft.

Ls-Dyna provides a variety of material models. To capture rate-dependent loading and hysteretic unloading of foams the material model *MAT_FU_CHANG _FOAM [54] is well suited. It is used for a wide variety of foam materials [55] and can be mapped with near test data. *MAT_FU_CHANG_FOAM requires as input data engineering stress vs. engineering strain as a function of multiple constant strain rates. The material model is characterized by a series of pendulum impact and quasi-static tests, which the authors conducted and elaborated in [35]. As summarized in Fig. 17b, for rate-dependent loading, the stress is approximated by a surface interpolation of the experimental test with different impact speeds $v_{1}$; for rate-dependent unloading, an approximation of the quasi-static loading $v_{\mathrm{const.}}$ and a parameterized damage formulation from LS-Dyna is used. The approximation is a bipolynomial regression by minimizing a quadratic objective function, with linear equality and inequality constraints incorporating LS-Dyna material card requirements and knowledge about surface topology.

### Rider surrogates

**Fig. 18:**
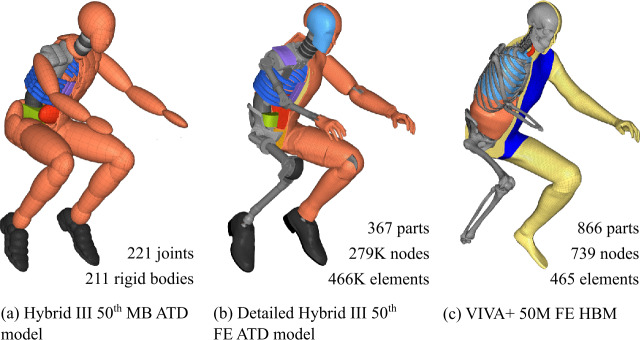
Models ((a): Madymo [56], (b): LSTC [57], (c): Project VIRTUAL [58, 59]) of anthropometric test devices and human body models as rider surrogates (partially blanked).

Since the workflow includes the two important simulation software environments of MBS and FE, the choice of numerical models for representing motorcyclists is broad. Thus, MB and FE models of ATDs as artificial mechanical human surrogates and virtual only human body models (HBMs) with a wide range of complexity and computational costs can be used. These are instrumented with sensors that measure accelerations, forces, moments, and deformations at multiple body locations. The selection for the results of this work consists of the models from Fig. 18 (excluding sex variants). The positioning of kinematically coupled MB models is particularly simple, fast, and, above all, user-friendly. The positioning of FE models is far more complex and time-consuming, i.e., laborious in pre-processing. For ATD models, overlapping parts and elements can occur. Here, this occurs at the thighs-to-pelvis connections of the molded hip flexion angle, the knee bends, where the shanks touch the thighs, and the ATD wrists. HBMs have complex joints and motion patterns with nonidealized degrees of freedom surrounded by deformable tissue. To position the models, transient seating simulation incorporates deformation and compliance between deformable parts. For a detailed description of the authors’ work on the positioning and contact initialization process of ATDs, see [33]; for HBMs, see [34].

To evaluate rider surrogate loading, injury criteria correlate sensor loads with probabilities of certain injuries for specific body regions. Since the severity of an injury cannot always be correlated with the maxima of the load, derived quantities, such as the head injury criterion (HIC) [60], have been developed. It is a normalized integral of the resultant of the acceleration $a_{\mathrm{res}}(t) = \|\boldsymbol{a}(t)\|_{2}$ measured at the head’s center of gravity, and it rates the loading by its value and duration by

7 $$ \mathrm{HIC}(t_{2}-t_{1})= \max _{t_{1},t_{2}}\left \lbrace \left (t_{2}-t_{1}\right )\left [\frac{1}{t_{2}-t_{1}} \int _{t_{1}}^{t_{2}} a_{\mathrm{res}}(t) dt\right ]^{2.5}\right \rbrace , $$

where $a_{\mathrm{res}}(t)$ is expressed in standard gravitational acceleration $g =9.81$ m/s^2^ and $t$ in s. Furthermore, a criterion that represents the maximum value over a certain period of time is often used, such as the $a_{\mathrm{3ms}}$-criterion of the resultant acceleration

8 $$ a_{\mathrm{3ms}}=\max _{t_{1}}\left (\min _{t_{1} \le t \le t_{1}+\mathrm{3ms}}a_{\mathrm{res}}(t)\right ). $$

The selection of injury criteria with recommended biomechanical thresholds considered are summarized in Table 1. It is based on a comprehensive set of injury criteria and corresponding biomechanical limits for motorcyclists from a literature review by [38].

**Table 1:** Evaluated injury criteria with biomechanical limits from [38] for motorcyclists

body region	injury criterion	abbreviation	limit
head	head injury criterion	HIC(36)	1000 for 36 ms
	resultant head acceleration	$a_{\mathrm{3ms}}$	80 g over 3 ms
neck	neck tensile force	$F_{z, \mathrm{1ms}}$	3.3 kN over 1 ms
		$F_{z, \mathrm{45ms}}$	1.1 kN over 1 ms
	neck compression force	$F_{-z, \mathrm{1ms}}$	4 kN over 1 ms
		$F_{-z, \mathrm{45ms}}$	1.1 kN over 1 ms
	neck shear force	$F_{xy, \mathrm{1ms}}$	4 kN over 1 ms
		$F_{xy, \mathrm{45ms}}$	1.1 kN over 1 ms
	neck rearward moment	$M_{y-, \mathrm{max}}$	190 Nm
	neck forward moment	$M_{y, \mathrm{max}}$	57 Nm
thorax	resultant thorax acceleration	$a_{\mathrm{3ms}}$	80 g over 3 ms
	chest deflection	$\mathrm{defl}_{\mathrm{max}}$	75 mm
pelvis	resultant pelvis acceleration	$a_{\mathrm{3ms}}$	80 g over 3 ms
femur	femur axial force	$\left \lvert F_{z} \right \rvert _{\mathrm{max}}$	10 kN

## Simulation results

**Fig. 19:**
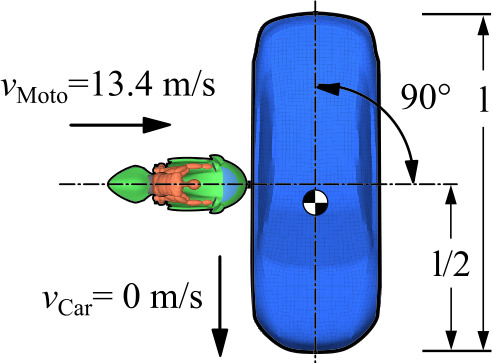
Frontal impact scenario according to ISO 13232 [61]

In the following, results from stages 1 to 3 for a frontal collision against a stationary passenger car equivalent to the full-scale crash test of the conventional motorcycle, see Fig. 19, are given.

### Stage 1: combined multibody and finite element model

**Fig. 20:**
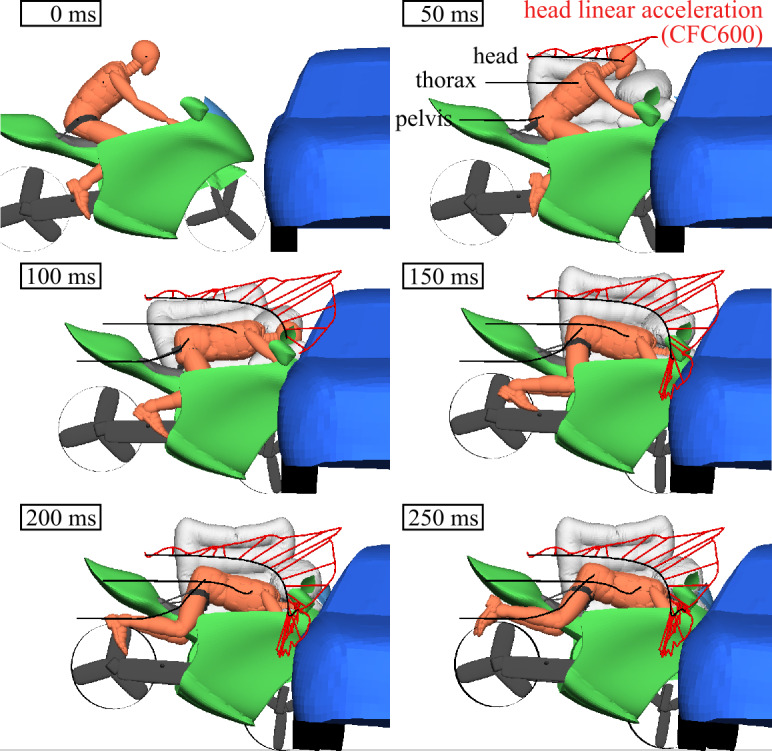
Impact trajectories (black) with head accelerations (red) in combined MB/FE simulations with Hybrid III 50^th^ ATD (Note: The right airbags are not displayed)

**Fig. 21:**
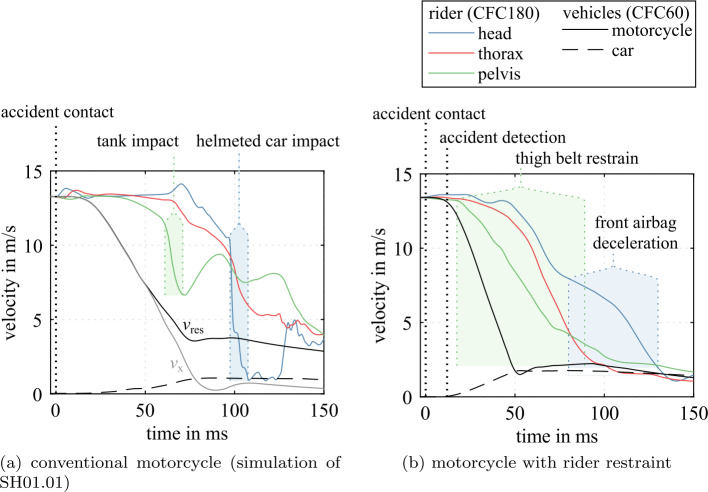
Vehicle and rider impact velocities in MB and combined MB/FE simulations

This stage is used to predict the impact dynamics of the vehicles and the rider’s interaction with the passive safety system, see Fig. 20. The frontal shape of the motorcycle with its voluminous cockpit fairing and overhanging nose act as a crash structure that allows for some deformation. This results in a much larger front fork deformation. This high contact point counteracts a rotational moment that would otherwise cause the motorcycle to rotate around its transverse/pitch axis. This leads to a stiffer impact response as shown by the faster velocity drop in Fig. 21. Also, because the accident trajectory of the motorcycle with safety concept and its rider results in less rotation and less upward motion, the energy transfer to the car is greater. As shown, the belts restrain the pelvis. Belt load limiting allows for some forward displacement of the rider. The failing attachment of the windshield allows for maximum forward displacement of the rider. The restraint leads to an upper body rotation with the head and upper torso, consequently impacting the frontal airbag. The pressure regulation of the airbags through exhaust holes prevents a rebound of the rider. The overlay of head linear acceleration shows that a maximum is reached during deceleration from the belt restraint, not when impacting the airbag.

Figure 21 shows the velocities of the vehicles and the main body parts of the rider for the conventional motorcycle impact (a) and the motorcycle with proposed rider restraint (b). In the conventional impact, the rider is decelerated abruptly by the pelvis impacting the tank and the helmeted head impacting the car—the restrained rider is decelerated more continuously over a more extended time period. Simulations of the novel motorcycle concept without its safety equipment would not be an adequate benchmark for evaluating its benefits. For conventional motorcycles, it is considered advantageous that a motorcyclist does not become entangled in parts of the motorcycle body and instead separates from the motorcycle as soon as possible [38]. The proposed body is not designed to allow such accident kinematics.

**Fig. 22:**
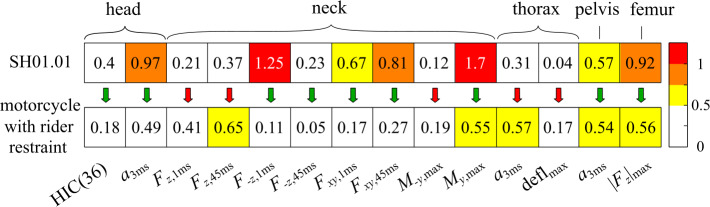
Injury criteria relative to the respective biomechanical limit for the conventional motorcycle (top; experimental data of SH01.01) and the motorcycle with rider restraint (bottom; stage 1 simulation)

In Fig. 22, the resulting injury criteria are plotted relative to their biomechanical limits for the real-world crash test versus the MB simulation of the restraint safety concept. In conventional accidents, the head hits the roof rail. This leads to high head accelerations, a critical compression of the cervical spine with a very high peak rearward neck moment. For the impact with safety concept, the highest sensed body loads are the head, thorax, and pelvis accelerations and neck tensile forces. These loads depend on the implemented belt load limit selected based on a trade-off between tolerable body loads and acceptable forward body displacement. Compared to the conventional crash impact body loads, most criteria are reduced, lower overall, and within their respective limits.

### Stage 2: prescribed finite element rider interaction model

**Fig. 23:**
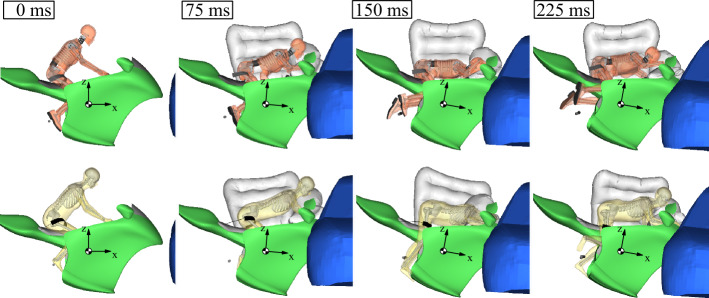
Impact responses of Hybrid 5^th^ ATD (top) and 50F ViVA+ HBM (bottom) in coupled FE/MB simulations (Note: The right airbags are not displayed)

The modeling of the impact dynamics in the FE environment is used to investigate more rider surrogate models while focusing on the interaction behavior with the safety systems. ATDs are not applicable omnidirectional. Instead, they are specific for different load directions and postures and are most often designed to depict occupants of automobiles. The stage 2 simulation environment is well suited to study state-of-the-art HBMs. Figure 23 shows a comparison of the kinematic impact response of a 5^th^ Hybrid III ATD with a female 50^th^ VIVA 50F HBM. Overall, the impact kinematics turn out very similar. With the HBM, it can be observed that the belt slips down at 225 ms. This could possibly be prevented with other belt variants. In evaluating biomechanical injuries, the capabilities of the HBMs go far beyond those of mechanical surrogate models and their virtual representation. Here, HBMs provide much more profound insight. They, e.g., allow observing strain-based human injury mechanisms.

The energy balance of each part or a subset of parts in LS-Dyna is the sum

9 $$\begin{aligned} E_{\mathrm{total}}(t) = \; & E_{\mathrm{kinetic}}(t) + E_{ \mathrm{internal}}(t) + E_{\mathrm{potential}}(t) \\ & + E_{\mathrm{contact}}(t) + E_{\mathrm{sliding \; interface}}(t) + E_{ \mathrm{hourglass}}(t). \end{aligned}$$

**Fig. 24:**
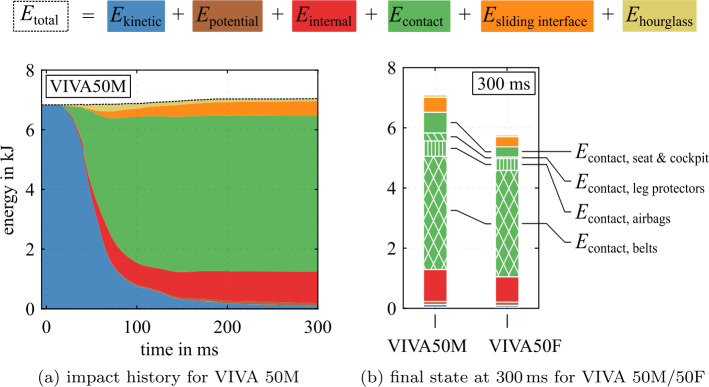
Rider energy balances in coupled FE/MB simulations

Figure 24 gives the energy balance of VIVA 50M and 50F HBMs during the impact. The kinetic, internal, and potential energies represent the rider’s current energy level. The contact energy is the energy transferred to external contact partners. The sliding interface energy is the stored energy of internal contacts, and the hourglass energy is an FEM-specific numerical quantity and should be minimal or at least very low. Figure 24a shows that initial energy is solely its initial kinetic energy, which is reduced to a minimum during impact. Most of it is transferred to the contact partners; some of it is converted to internal energy. The rider is decelerated through the application of forces. Anything that applies these forces can be considered part of the restraint system. Examining the contributors to the contact energy transfer in Fig. 24b, the cumulative belt contact energy transfer in the final state at 300 ms has the most significant share, followed by the airbags and the remaining cockpit surfaces. The leg impact protection has a comparably small share.

### Stage 3: full finite element model

**Fig. 25:**
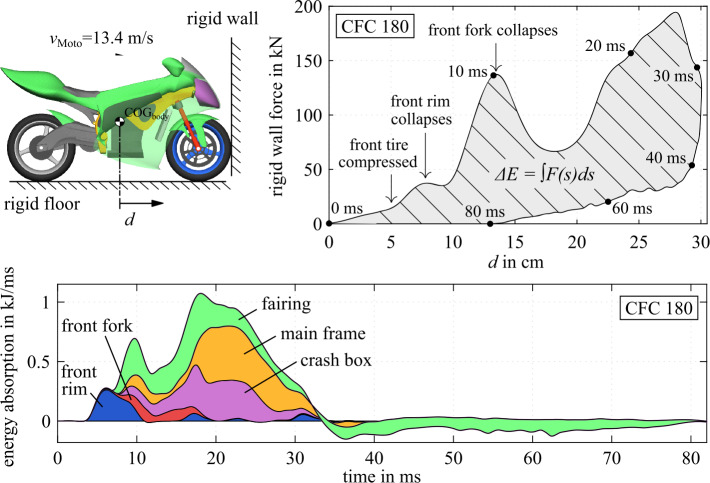
Frontal deformation characteristics of motorcycle (top) with stacked internal energy absorption of main structural motorcycle components (bottom) from full FE simulation of rigid wall impact

**Fig. 26:**
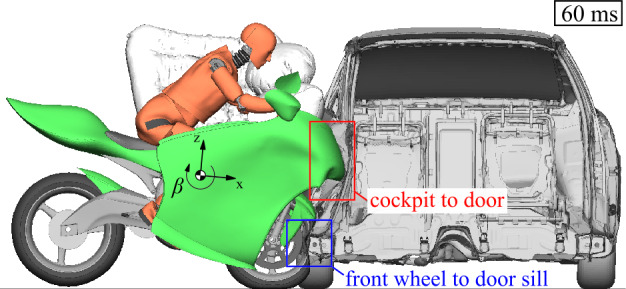
Motorcycle to car structural interaction and intrusion behavior in full FE simulation (Note: The right airbags are not displayed)

The full FE representation is used to provide (i) the deformation characteristics and vehicle strength of the motorcycle itself and (ii) the structural interaction between the motorcycle and the opposing vehicle. In Fig. 25 the distance $d$ after an impact with an initial velocity $v_{ \mathrm{Moto}}$ = 13.4 m/s is given. The plot of distance $d$ against the resulting rigid wall force shows several phases of impact deformation, which can be split into compression of the front tire, followed by the collapse of the front wheel rim, and then the collapse of the front fork. The evaluation of the internal energy absorption (initial dissipation and comparably low elastic release) of the main structural components involved shows that the impact energy during the subsequent deformation phase until maximum compression is dissipated by the crash box, main frame, and fairing of the motorcycle. Since these all act at a high point of contact, this counteracts pitching of the motorcycle. The structural interaction and intrusion behavior between the motorcycle and the opposing vehicle is shown through a section cut of the opposing car in Fig. 26. The front wheel of the motorcycle collides with the car’s passenger side front door sill. The motorcycle cockpit deforms the car’s door inwards, while the motorcycle’s cockpit itself is little deformed. The deformation of the telescopic front fork is, by design, much greater than on the conventional motorcycle discussed here. This analysis shows that the very rigid sill structure promotes motorcycle pitching. Nevertheless, the motorcycle does not roll over. The maximum pitching angle in this simulation is $\beta (201~\mathrm{ms}) = 15.1^{\circ}$.

## Discussion

Models with varying levels of detail and complexity have been developed. The models have modular input decks for the restraint systems, rider surrogate models, and opposing (vehicle) structures. Custom translators are used for the exchange between the software environments, which transfer, e.g., scenario parameters and FE-meshes (particularly airbag geometries) from design iterations. Each of the model and simulation environments allows for specific investigations with different simulation runtimes, see Table 2. They have individual advantages and are suitable for assessing and optimizing various aspects of the accident behavior of a motorized two-wheeler. The first stage MB approach features low complexity and low numerical costs while capturing the essential physics of the collision. This represents a numerically efficient way to tune and improve the safety system, i.e., adapting the properties, shapes, and locations of the safety components. The third stage full FE approach aims to fully represent the interaction of the collision to accurately predict the performance of the finalized design by replicating every structural component of the vehicles in great detail. However, this is bought by a challenging model generation and significantly increased computational efforts. The advantages of the second stage partial FE approach using MB-vehicle interaction of the numerical strategy presented here are the successive methodological model generation and gradually increased level of detail and expected fidelity while significantly reducing computation time compared to a full FE model representation. This hybrid model is comparable to already realized approaches for occupants in cars, see, e.g., [19]. This offers the possibility to consider a larger variance of accident scenarios or occupant diversity or to enable very complex and numerically expensive investigations with FE human body models at reduced numerical complexity. To exploit the full range of existing mathematical modeling in the area of crash simulation, reduced order models and response-surface models must also be used to optimize aspects with numerical methods at reduced computational costs.

**Table 2:** Solver run times for an impact scenario with 300 ms (AMD Ryzen 9 5950X 16-Core CPU@3.4GHz)

stage 1:	stage 2:	stage 3:
combined MB/FE model	coupled FE/MB model	full FE model
w/ Hyb III 50^th^ ATD:	w/ Hyb III 50^th^ ATD:	w/ Hyb III 50^th^ ATD:
**36 min/16 CPUs (SMP)**	**10 h/16 CPUs (MPP)**	**59.3 h/16 CPUs (MPP)**
	w/ VIVA 50M HBM:	
	**23.3 h/16 CPUs (MPP)**	

SMP: shared memory processing

MPP: massively parallel processing

Predicting the accident outcome of such a complex interaction is a complex task. At this stage, the MB model has shown that it can predict the vehicle interaction between a conventional motorcycle and a passenger car. Real-world performance of the safe motorcycle has not yet been demonstrated, and the models are not currently validated by experiments. Nevertheless, the work shows what is possible with a solely simulation-driven approach. This will be necessary in the future to quickly develop innovative solutions with minimal funding to investigate a new approach conceptually quickly.

## Conclusions

*RQ1:* In a frontal collision scenario against a stationary opponent, in this case a passenger car, the simulations of the restraint system show its effect of a guided and controlled trajectory and deceleration of the motorcycle rider. It consists of (i) restraining the rider’s pelvis to the motorcycle, (ii) decelerating the resulting upper and lower body motion by airbags and leg impact protectors, and (iii) preventing hard contact with opposing structures. This results in fewer critical biomechanical loads on the rider. There may be other load cases where detrimental effects result from the rider restraint. To thoroughly answer the question, investigations of additional scenarios that include behavior in subsequent secondary accident phases after an initial impact, including solo accidents, are necessary.

*RQ2:* The shown numerical research strategy outlines a novel procedure in virtual motorcycle accident research and passive safety equipment development. It is a systematic model generation approach with different levels of computational effort and model complexity. It demonstrates a meaningful combination of current state-of-the-art MB and FE simulation software environments to model the same problem with method-specific advantages and disadvantages. The strategy enabled the virtual design and dimensioning of a novel safety system with little time and resources and aimed for a step-by-step validation of individual components in the future. Although the procedure is presently applied for a motorcycle with a novel restraint safety concept, applying similar procedures in virtual research for conventional (powered) two-wheelers is highly desirable.
